# Fiber Bragg Grating Pulse and Systolic Blood Pressure Measurement System Based on Mach–Zehnder Interferometer

**DOI:** 10.3390/s24196222

**Published:** 2024-09-26

**Authors:** Yuanjun Li, Bo Wang, Shanren Liu, Mengmeng Gao, Qianhua Li, Chao Chen, Qi Guo, Yongsen Yu

**Affiliations:** 1 State Key Laboratory of Integrated Optoelectronics, College of Electronic Science and Engineering, Jilin University, Changchun 130012, China; liyj3422@163.com (Y.L.); wbo22@mails.jlu.edu.cn (B.W.); liusr21@mails.jlu.edu.cn (S.L.); gaomm23@mails.jlu.edu.cn (M.G.); qhli24@mails.jlu.edu.cn (Q.L.); 2 State Key Laboratory of Luminescence Science and Technology, Changchun Institute of Optics, Fine Mechanics and Physics, Chinese Academy of Sciences, Changchun 130033, China; chenc@ciomp.ac.cn

**Keywords:** fiber Bragg grating, edge filter, pulse, systolic blood pressure

## Abstract

A fiber Bragg grating (FBG) pulse and systolic blood pressure (SBP) measurement system based on the edge-filtering method is proposed. The edge filter is the Mach–Zehnder interferometer (MZI) fabricated by two fiber couplers with a linear slope of 52.45 dBm/nm. The developed system consists of a broadband light source, an edge filter, fiber Bragg gratings (FBGs), a coarse wavelength-division multiplexer (CWDM), and signal-processing circuits based on a field-programmable gate array (FPGA). It can simultaneously measure pulse pulsations of the radial artery in the wrist at three positions: Cun, Guan and Chi. The SBP can be calculated based on the pulse transit time (PTT) principle. The measurement results compared to a standard blood pressure monitor showed the mean absolute error (MAE) and standard deviation (STD) of the SBP were 0.93 ± 3.13 mmHg. The system meets the requirements of the Association for the Advancement of Medical Instrumentation (AAMI) equipment standards. The proposed system can achieve continuous real-time measurement of pulse and SBP and has the advantages of fast detection speed, stable performance, and no compression sensation for subjects. The system has important application value in the fields of human health monitoring and medical device development.

## 1. Introduction

Hypertension has always been one of the common chronic diseases that pose a threat to human health. Blood pressure monitoring plays an important role in preventing cardiovascular diseases. In traditional blood pressure measurement, an inflatable cuff is usually used. In this method, the cuff is fixed to the upper arm of the subject and the artery is compressed by inflating the cuff, using a sensor to measure the pressure of the artery. However, when the cuff is tightened, it can cause a sensation of compression for the subjects and make them feel uncomfortable. Continuous non-invasive measurement of blood pressure can be achieved based on the principle of the pulse transit time (PTT) [1,2]. The principle is to collect the corresponding arterial signals by placing the sensors at different positions from the heart; the time difference between the characteristic points of the pulse wave signal is used to further calculate the blood pressure. At present, photoplethysmography (PPG) is widely used for pulse measurement [3]. PPG is a non-invasive monitoring method that uses photoelectric means to monitor changes in blood volume within living tissue. When a certain wavelength of light is irradiated to the skin of the fingertip, the contraction and expansion of blood vessels will affect the transmission intensity or reflection intensity of light during each heartbeat. The change in light intensity reflects the pulsation state of the artery. However, the light source intensity used in this method is generally weak and the collected signal is affected by the roughness and humidity of the fingertip skin, which will decrease the signal-to-noise ratio [4].

In recent years, fiber Bragg grating (FBG) sensors have attracted more and more attention from researchers. They have the advantages of small size, light weight, and high sensitivity. They are widely used in bridge monitoring [5,6], oil pipeline monitoring [7,8], power system monitoring [9,10,11], physiological information monitoring [12], and other fields. The FBG is a kind of wavelength-modulation fiber device. When the broadband light passes through the FBG, the light that meets the resonant condition is reflected, while the rest continues to transmit. By detecting the change in reflected wavelength, external physical quantities such as temperature and strain can be reflected.

The strain sensor based on the FBG can achieve real-time pulse detection with high sensitivity [13]. FBG uses the principle of strain to measure pulse without interference from human skin color and fingertip skin humidity. The ASE broadband light source used in the FBG sensing system can provide a high output power, which can avoid the problem of the low signal-to-noise ratio of photodetectors in detecting weak light signals in PPG methods. The pulse pulsation is the conduction of blood pulses through the arteries to the skin surface caused by the contraction of the heart. This process creates periodic mechanical stress on the skin and superficial tissues [14]. When the FBG sensor is attached to a pulse point such as the wrist or carotid artery, the small mechanical stress caused by pulse pulsation can result in the FBG wavelength drift. By accurately measuring the drift of this wavelength, information such as the frequency and intensity of pulse pulsation can be obtained. Due to the small changes in the FBG wavelength, it is usually necessary to use a high-sensitivity optical spectrum analyzer (OSA) or interrogator. By analyzing and processing the detected spectral data, characteristic parameters of the pulse can be obtained, such as heart rate, pulse waveform, etc. Due to the slow scanning speed, large size, and high cost of the OSA, it cannot quickly process the scanned data in real time [15]. It is not suitable for collecting and processing the real-time pulse signal.

The FBG interrogator is designed for real-time measurement. It is suitable for detecting high-frequency strain signals, such as pulses or other dynamic signals. However, the signal-processing algorithm and software of commercial interrogators are generally universal, which means they cannot effectively extract and process the key information in pulse signals, such as heart rate and pulse waveform. Therefore, it is necessary to research and design a more suitable FBG interrogation device for pulse and blood pressure detection.

In 2018, Jia et al. proposed a novel optical sensor for radial pulse measurement based on the FBG and lever amplification mechanism. They analyzed the relationship between the sensitivity of the sensor and the ratio of the lever arm. The sensitivity of the FBG sensor can reach 8.236 nm/N [16]. In 2019, Koyama et al. used plastic optical fiber to measure pulse. After processing the pulse signal, a calibration curve was constructed by partial least squares regression. Then, blood pressure was calculated according to the calibration curve. The result showed that the signal-to-noise ratio of the pulse signal measured by plastic optical fiber was more than eight times higher than silica fiber [17]. In 2021, Shi et al. proposed a novel FBG-based hybrid force and displacement sensor. It can restore the radial waveform with high precision and achieve force and displacement resolution values of 0.47 mN and 0.103 μm [18]. In 2021, Asokan et al. reported a non-invasive blood pressure monitoring method based on FBG sensors. The radial arterial pulse waveform was acquired by the Fiber Bragg Grating-based Pulse Monitoring Device. The waveform was processed to obtain the blood pressure value. Compared with the standard measuring instrument, the measurement error of this device was less than 10 mmHg [19].

A fiber Bragg grating pulse and systolic blood pressure (SBP) measurement system based on the Mach–Zehnder interferometer (MZI) is proposed in this work. The edge slope of the MZI is 52.45 dBm/nm. The system is composed of a broadband light source, fiber Bragg gratings (FBGs), an edge filter, a coarse wavelength-division multiplexer (CWDM), and signal-processing circuits based on a field-programmable gate array (FPGA). The collected electrical signals were sent to the Labview 2021 for processing through the Ethernet User Datagram Protocol (UDP). Three FBG sensors were inscribed using femtosecond laser, and pulse pulsations were measured at the wrist positions called Cun, Guan, and Chi simultaneously. By fixing two FBGs at the position of the left and right radial arteries, the SBP was measured using the PTT principle. We compared the measurement results with those of a standard electronic blood pressure monitor and found that the mean absolute error (MAE) and standard deviation (STD) of the SBP were 0.93 ± 3.13 mmHg. The proposed system has stable performance, compact structure, fast detection speed, and no compression sensation for the subjects. It can use one MZI edge filter to interrogate multiple FBGs simultaneously. Real-time monitoring of the pulse and systolic blood pressure can be achieved. The many advantages make this measurement system extremely promising for vital sign monitoring and medical device development.

## 2. Principle of the System

### 2.1. Principle of Optical Path Design

The FBG is a fiber structure that can reflect light of a specific wavelength. When light is incident on the FBG, the light of the specific wavelength that matches the grating period will be reflected, while the rest will continue to propagate. From the phase-matching condition, it can be concluded that [5,20]

(1) $$m\lambda_{B}=2n_{eff}\Lambda$$

where $\lambda_{B}$ is the FBG central wavelength, $n_{eff}$ is the effective refractive index of the fiber core, Λ is the grating period, and m is the grating order. When the FBG is affected by strain, the effective refractive index and grating period of the grating region will be changed, which will cause the FBG central wavelength to change. By detecting the wavelength change of the reflected light, the strain can be accurately detected.

**Figure 1:**
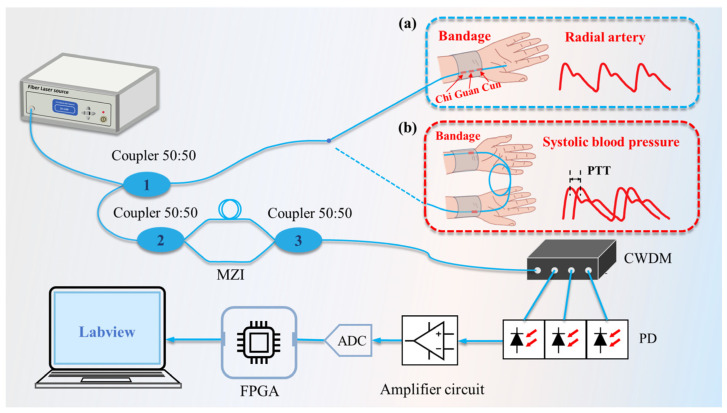
Schematic diagram of pulse and systolic blood pressure (SBP) measurement system. (**a**) Schematic diagram of pulse measurement; (**b**) Schematic diagram of SBP measurement.

At present, there are various interrogation methods for the FBG, including Sagnac interferometry [21,22], matched grating filtering [23], microwave spectrum analysis [24], edge filtering [25,26,27], etc. The matching grating method requires a high-precision mechanical positioning system to achieve wavelength matching between the reference grating and the sensing grating, which increases the complexity of the system. The Sagnac interferometry method requires complex signal-processing algorithms to interrogate the phase changes of the optical signal, which increases the difficulty of signal demodulation. Compared to the above two methods, edge filtering has the advantages of simple structure, good stability, and lower cost. Within a certain range, edge filtering can achieve a good linear response. The edge-filtering method usually adopts a narrowband filter and can respond quickly to wavelength changes, so it is suitable for application scenarios that require real-time monitoring. In this work, an edge-filtering method based on the MZI is used to monitor pulse and SBP in real time. The schematic diagram of the whole measurement system is shown in Figure 1.

The diagrams of the pulse and SBP measurement method are shown in Figure 1a,b. The FBGs were attached to an elastic bandage using double-sided tape. The elastic bandage was placed over the wrist and the FBGs were aligned to the radial artery to detect the pulse. The light emitted by the amplified spontaneous emission (ASE) broadband light source was incident on the FBGs through coupler 1, and the reflected light was split by coupler 2 after passing through coupler 1 and interfered in coupler 3. The output power of the ASE broadband light source is 5 mW. CWDM separated the reflected light of FBGs with different wavelengths. The optical signals were detected by photodiodes. The signals were further processed through the FPGA hardware circuits and Labview 2021.

The MZI is an optical device that uses two beams of light that pass through different paths and overlap together to achieve measurement through the interference effect. The MZI can be designed as an edge filter, meaning that its transmission spectrum has steep edges near a certain wavelength. This means that when there is a small change in wavelength, the transmitted light intensity will undergo a significant change.

**Figure 2:**
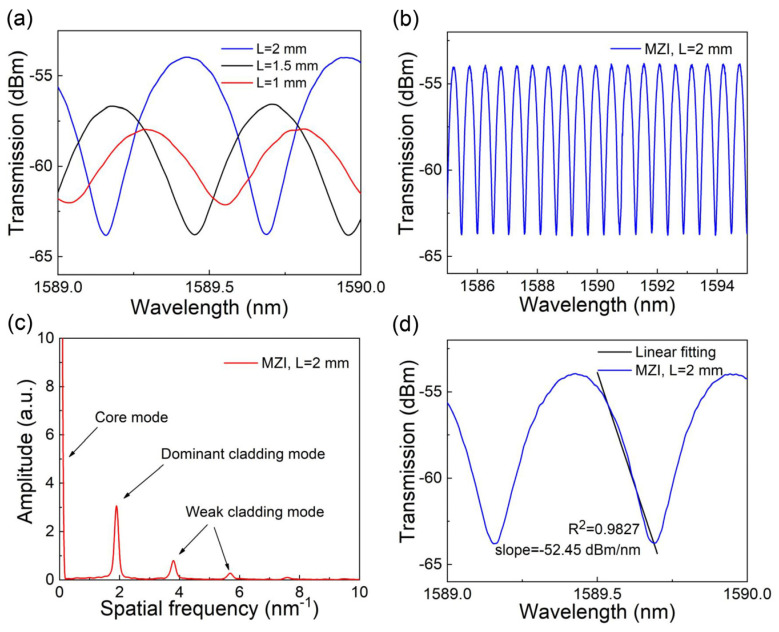
(**a**) Interference spectra of the Mach–Zehnder interferometers with varying arm length differences; (**b**) interference spectrum of the Mach–Zehnder interferometer (MZI) with arm length difference of 2 mm; (**c**) spatial frequency spectrum of the MZI with arm length differences of 2 mm; (**d**) linearity of the MZI in the range of 1589.45–1589.70 nm.

The MZI in this system consists of two 3 dB couplers, and the interference spectrum is a spectral line that approximates a cosine function. According to the interference principle, the interference intensity can be expressed as [28]

(2) $$I=I_{1}+I_{2}+\sqrt{I_{1}I_{2}}\cos\Delta \varphi$$

where $I_{1}$ and $I_{2}$ are the intensity of the optical signals from the two interference arms, and Δφ is the phase difference between the optical signals from the two interference arms, which can be expressed as

(3) $$\Delta \varphi =\frac{2\pi \Delta n_{eff}L}{\lambda }$$

where $\Delta n_{eff}$ is the effective refractive index difference between the two interference paths, L is the arm length difference of the interference arms, and λ is the wavelength of the incident light. The falling edge in the interference spectrum is selected as the filtering edge. When the light reflected by the FBG passes through the falling edge, the output intensity of the filter will change, which can be expressed as [29]

(4) $$\Delta I(\Delta \lambda_{B})=kR_{0}\sqrt{\frac{\pi }{a}}\Delta \lambda_{B}$$

where k is the slope of the linear edge filter, $\lambda_{B}$ is the FBG center wavelength, $R_{0}$ is the peak reflectivity, and a is a constant related to the FBG. It can be seen that there is a linear relationship between the change in light intensity and the wavelength change of the FBG. When the FBG wavelength changes, the voltage output by the photodetector will also change. In order to obtain a better interference spectrum, the interference spectra of Mach–Zehnder interferometers with varying arm length differences were investigated, and their interference spectra are shown in Figure 2a. In the experiment, the MZI with an arm length difference of 2 mm was selected as the edge filter, and the MZI interference spectrum is shown in Figure 2b.

A Fourier transform was performed on the interference spectrum of the MZI, and the obtained spatial spectrum is shown in Figure 2c. The results showed that the peak intensity was strongest at point zero and the core mode was dominant. There was also one dominant cladding mode and other weak cladding modes. The amplified interference spectrum is shown in Figure 2d, where the edge slope reached 52.45 dBm/nm in the range of 1589.45–1589.70 nm. The pulses of different people were measured using the FBG sensor and the wavelength drift of the FBG was no more than 80 pm. This is similar to previous research [30]. Therefore, the MZI with an arm length difference of 2 mm was chosen. It has a linear range of about 250 pm. The range of this linear region allows the FBG wavelength to remain in the linear region when it fluctuates dynamically.

When stress is applied to the FBG, the Bragg wavelength will change. The wavelength reflected by the FBG will pass through the MZI edge filter, resulting in a change in the output light intensity of the filter. Since the spectral edge of the filter is very sensitive to wavelength changes, the FBG wavelength drift can be accurately measured by monitoring the change in the output light intensity. By comparing the measured light intensity change with the pre-calibrated transmission characteristic curve of the MZI filter, the wavelength change of the FBG can be deduced and the sensing signal can be interrogated.

### 2.2. Principle of Circuit Design

**Figure 3:**
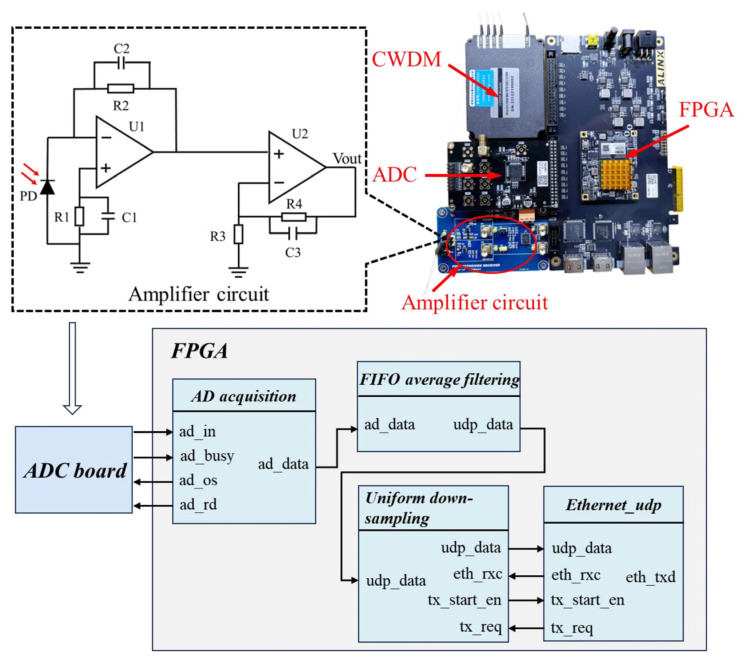
Principle and physical diagram of the photoelectric amplifier circuit and a field-programmable gate array (FPGA) module.

CWDM was used to separate the reflected light of FBGs with different wavelengths, and the interference light signal was collected by a photodiode (PD, LSIPD-A40-B-SMFA, Lightsensing, Beijing, China), as shown in Figure 3. The spectral response range of the PD is 800–1700 nm, with a responsivity of 0.85 A/W around the wavelength of 1550 nm. A front-end trans-impedance amplifier circuit based on AD8065 (AD7606, ADI, Norwood, MA, USA) was designed to achieve the conversion of photocurrent to voltage. To facilitate high-precision signal acquisition by subsequent circuits, the operational amplifier LM6144 (AD7606, ADI, Norwood, MA, USA) was used to amplify the voltage signal twice. The voltage signals were collected through an eight-channel ADC (AD7606, ADI, Norwood, MA, USA) and further processed through FPGA (Artix-7, Xilinx, San Jose, CA, USA).

To suppress circuit noise and power frequency interference, a first-in-first-out (FIFO) average filtering algorithm module was installed inside the FPGA. This module stored the collected data in FiFo, and when 48 data points were accumulated in FiFo, the average of all data points was calculated. FiFo followed the principle of first-in-first-out, where the data stream was continuously updated to achieve real-time average filtering. The data, after average filtering, were sent to the uniform down-sampling module. By extracting data at equal intervals, the uniform down-sampling module controlled the amount of data within the range that could be sent by UDP. The pulse data sent to the computer were processed by the Labview 2021 program, which used a Butterworth bandpass filter to filter out baseline drift and high-frequency noise components of the original signal. Considering the frequency range of the pulse, the high-cutoff frequency was set to 10 Hz and the low-cutoff frequency was set to 0.3 Hz. The optical signal modulated by the pulse was collected from the hardware circuit and processed by the Labview 2021. The pulse waveforms were restored with high precision.

## 3. Experiment and Discussion

### 3.1. Pulse Measurement

The pulse reflects the frequency and intensity of the heartbeat. By regularly monitoring the pulse, potential health problems can be detected early and treatment can be carried out in a timely manner. Pulse diagnosis is also an important diagnostic method in traditional Chinese medicine (TCM) treatments. In TCM diagnosis, the health of the human body can be felt by pressing three positions called Cun, Guan and Chi at the wrist with three fingers. The position of the styloid process of the radius is called Guan, the position before Guan is called Cun, and the position after Guan is called Chi [31]. Pulse diagnosis plays an important role in providing treatment guidance and monitoring conditions in TCM. In this experiment, three FBGs were cascaded to simultaneously measure the pulse status at the three positions of Cun, Guan, and Chi.

**Figure 4:**
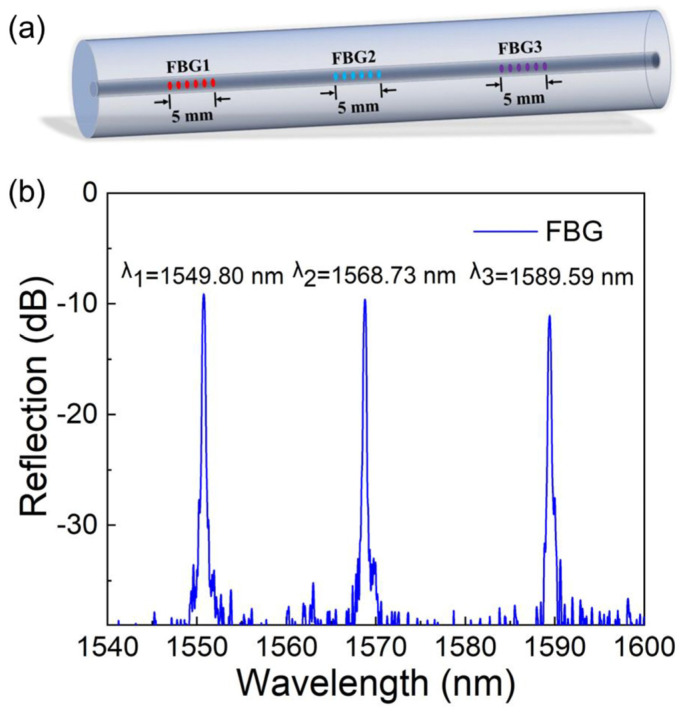
(**a**) Distribution diagram of fiber Bragg gratings (FBGs); (**b**) reflection spectra of FBGs.

Three FBGs were inscribed in a single-mode fiber (SMF-28e, Corning Inc., Corning, New York, NY, USA) using femtosecond laser. The lengths of the three FBGs were designed to be 5 mm and the distance between the adjacent FBGs was 15 mm. The central wavelengths were 1549.80 nm, 1568.73 nm, and 1589.59 nm, respectively. The center wavelength of the designed FBGs was located in the middle of the falling edge of the MZI transmission spectrum. The distribution diagram and reflection spectrum of the FBGs are shown in Figure 4a,b.

**Figure 5:**
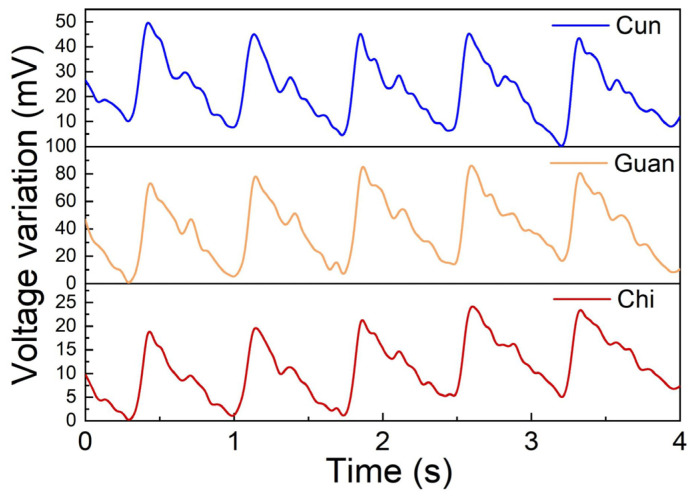
Pulse waveforms measured by the cascaded FBGs at the positions of Cun, Guan, and Chi.

We pasted three FBGs onto an elastic bandage using polyethylene terephthalate (PET) double-sided tape and then placed the elastic bandage over the right wrist. The three FBGs corresponded to Cun, Guan, and Chi. Five consecutive pulse waveforms from these three positions were collected simultaneously and the measured pulse waveforms are shown in Figure 5.

**Figure 6:**
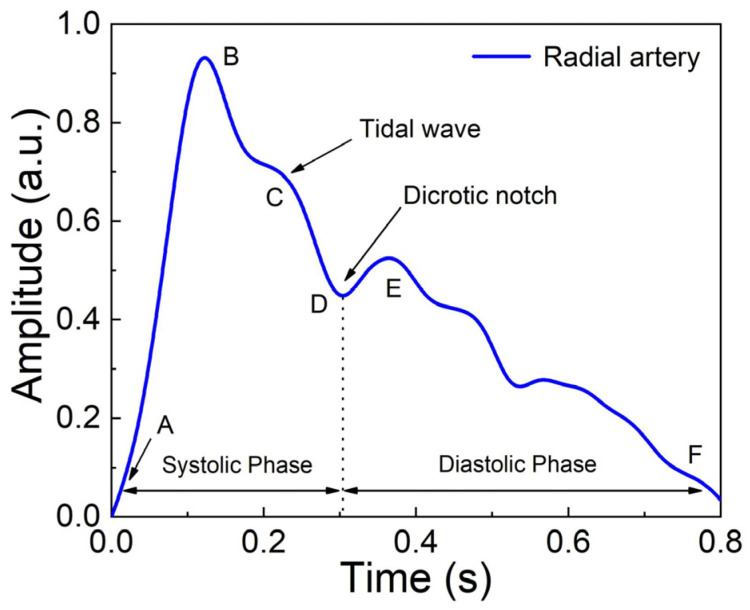
The pulse waveform of a single cycle collected by the system. (A: Starting point of pulse wave; B: Crest; C: Tidal wave; D: Dicrotic notch; E: Dicrotic wave; F: End point of pulse wave).

In order to further display the details of the pulse wave collected by the system, the pulse wave of a single period was analyzed. In the pulse wave diagram in Figure 6, the AB segment is called the ascending branch, and when the left ventricle contracts, blood is pumped into the artery and the pressure rises rapidly. The steepness of the waveform is related to arterial compliance and the contractility of the heart. The highest point of the pulse wave, point B, is called the crest, and the height and shape of the crest can reflect the function of the heart and the state of the arteries. Subsequently, the pressure in the arteries gradually decreases. The shape and inclination of the descending branch can reflect vascular resistance and compliance. As the arterial blood pressure gradually decreases, the blood from the aorta tends to flow back toward the ventricle, forming a tidal wave where point C is located. The retraction of the arterial wall causes the descending branch to descend rapidly and form a dicrotic notch. The dicrotic notch represents the moment the aortic valve closes, marking the end of the systolic phase of the heart and the beginning of the diastolic phase. The returning blood is blocked by the closed aortic valve, forming a dicrotic wave at point E. Subsequently, the blood pressure gradually decreases to point F. The results show that the system can accurately reproduce the details of human pulse waveforms and can be used for high-precision pulse acquisition.

### 3.2. SBP Measurement

Blood pressure refers to the lateral pressure exerted on a unit area of the vascular wall by blood flowing within an artery. By measuring and analyzing the pulse, blood pressure values can be indirectly measured based on the PTT principle. PTT refers to the time difference between pulse signal feature points located at different positions from the heart. According to the propagation formula of ideal fluid in an elastic cavity [32],

(5) $$PWV=K\sqrt{\frac{Eh}{\rho D}}$$

where PWV is the pulse wave velocity, which is inversely proportional to PTT at a certain propagation distance, *D* is the inner diameter of the vessel in equilibrium, *K* is the Moens constant, ρ is the blood density, and *E* is the Young’s modulus of the vessel wall, where

(6) $$E=E_{0}e^{\gamma P}$$

where $E_{0}$ is the elastic modulus of the blood vessel wall when the pressure is zero in a relaxed state, γ is a coefficient depending on the characteristics of blood vessels, and P is the arterial blood pressure value. Without considering some arterial parameters that have a small impact on blood pressure values, the expression for SBP can be obtained through the above formula as follows:

(7) $$SBP=a\ln(\frac{1}{PTT})+b$$

where a and b are undetermined coefficients. PTT values under different blood pressures are collected, and the corresponding PTT values and blood pressure values are linearly fitted to further obtain the values of a and b.

**Figure 7:**
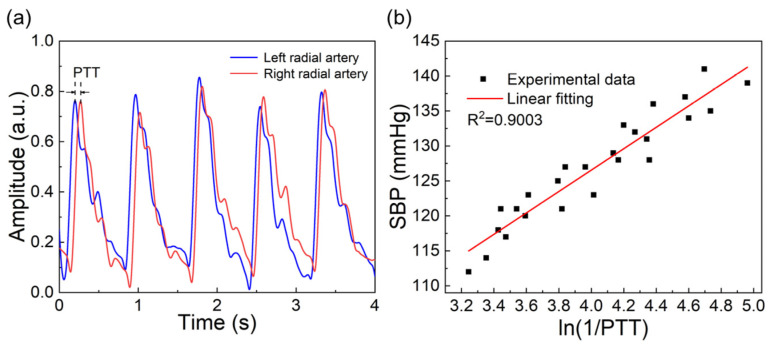
(**a**) Radial artery waveforms of left and right hands of subjects; (**b**) relationship between SBP and ln (1/PTT).

There are multiple arterial measurement points on the surface of the human body, such as the neck, wrist, ankle, etc. For the convenience of testing, the radial artery positions on the left and right hands of the tester subjects were selected as the testing points. We attached two FBGs onto each elastic bandage using PET double-sided tape and then the elastic bandages were placed on the left and right wrists of the subjects. The FBGs corresponded exactly to the position of the radial artery. Five people were selected as the research subjects in the experiment. During the experiment, the subjects were required to sit on a chair with their upper body upright. An electronic blood pressure monitor (HEM-7137, OMRON Corporation, Kyoto, Japan) was used to measure the SBP of the subjects, which was used as a reference value. Then, the pulses of the subjects were collected through the pulse measurement system in this experiment. Through the Peak Detection function in Labview 2021, the acquisition time corresponding to the pulse peak was calculated. To reduce experimental errors, five consecutive PTT values were taken as a group and their average value was taken as one data point. The collected pulse waveforms are shown in Figure 7a. It is worth mentioning that compared with the time difference between two wrists, the time difference between pulse peaks at different positions on one wrist can be ignored. We found that there was no significant difference in the calculation of PTT values when measuring different positions of the wrist during the experiment.

**Figure 8:**
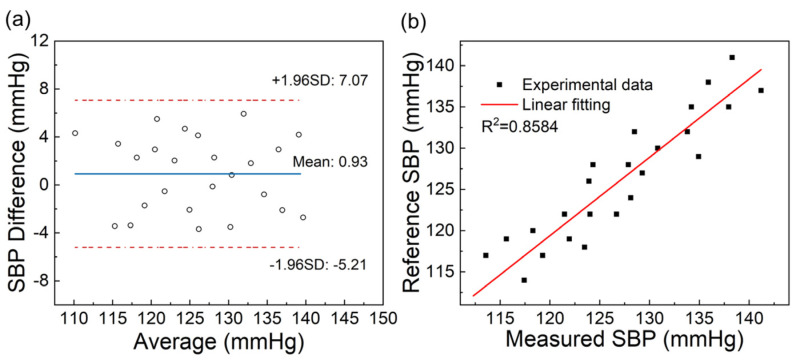
(**a**) Bland-Altman plot of measured and reference values of SBP; (**b**) relationship between measured SBP and reference values.

A total of 50 groups of data were collected in one day, among which 25 groups were used for experimental data fitting, as shown in Figure 7b. The results showed that SBP had a good correlation with ln(1/PTT), and the linearity reached 0.9003. The remaining 25 groups of data were used for experimental verification. In Figure 8a, the Bland-Altman plot was used to assess the consistency of the measured values with the reference values. The MAE and STD were 0.93 ± 3.13 mmHg. Linear regression was used to analyze the relationship between the measured values and the reference values, as shown in Figure 8b, and the correlation coefficient reached 0.8584. According to the standards of the Association for the Advancement of Medical Instrumentation (AAMI) [2], the MAE and STD between the SBP values collected by the SBP measurement system and those measured by the standard electronic blood pressure monitor are less than 5 mmHg and 8 mmHg, respectively. Therefore, the accuracy of the SBP measurement system meets the AAMI standard.

## 4. Conclusions

In conclusion, a multipoint pulse measurement system using the MZI as an edge filter has been proposed. The MZI featuring an edge filter slope of 52.45 dBm/nm was fabricated by fusing two 3 dB fiber couplers. The developed measurement system is implemented by a broadband light source, an edge filter, FBGs, and FPGA signal-processing circuits. It can simultaneously measure the pulse pulsation of the radial artery in the wrist at Cun, Guan, and Chi. The relationship between the time difference of pulse peaks in the left and right radial arteries and the SBP was explored, and the linearity of the fitted curve reached 0.9003. The Bland-Altman plot was used to evaluate the consistency between the calculated SBP and the reference values. The MAE and STD were 0.93 ± 3.13 mmHg, which meets the AAMI standards and verifies the high reliability and high precision of the measurement system. The proposed system can realize continuous pulse and SBP measurement and demonstrates potential for application in the domains of vital sign monitoring and medical device innovation. In future research, the system could be upgraded to a wireless transmission mode, which could further reduce the system size and improve system integration.

## Data Availability

The data presented in this study are available on request from the corresponding author.
